# Vascular events in viral hemorrhagic fevers: a comparative study of dengue and hantaviruses

**DOI:** 10.1007/s00441-014-1841-9

**Published:** 2014-03-13

**Authors:** Anon Srikiatkhachorn, Christina F. Spiropoulou

**Affiliations:** 1 Department of Medicine, University of Massachusetts Medical School, Worcester, MA USA; 2 Viral Special Pathogens Branch, Division of High-Consequence Pathogens and Pathology, Centers for Disease Control and Prevention, Atlanta, GA USA

**Keywords:** Endothelium, Viral hemorrhagic fevers, Dengue viruses, Hantaviruses, Permeability

## Abstract

Viral hemorrhagic diseases are a group of systemic viral infections with worldwide distribution and are significant causes of global mortality and morbidity. The hallmarks of viral hemorrhagic fevers are plasma leakage, thrombocytopenia, coagulopathy and hemorrhagic manifestations. The molecular mechanisms leading to plasma leakage in viral hemorrhagic fevers are not well understood. A common theme has emerged in which a complex interplay between pathogens, host immune response, and endothelial cells leads to the activation of endothelial cells and perturbation of barrier integrity. In this article, two clinically distinct viral hemorrhagic fevers caused by dengue viruses and hantaviruses are discussed to highlight their similarities and differences that may provide insights into the pathogenesis and therapeutic approach.

## Introduction

Four families of viruses are known to cause viral hemorrhagic diseases (VHFs): Flaviviridae, Bunyaviridae, Arenaviridae and Filoviridae. These viruses are distributed worldwide and are significant causes of global mortality and morbidity. The severity of VHFs varies widely from minor bleeding to fatal hemorrhagic fever characterized by plasma leakage and bleeding, which may lead to circulatory failure. Distinct vascular beds are involved in these diseases, resulting in different clinical manifestations. Despite these differences, viral hemorrhagic fevers share common clinical and laboratory findings, such as plasma leakage, hemoconcentration, thrombocytopenia and coagulopathy. These similarities point to a pathophysiological process shared by these conditions.

Since plasma leakage and bleeding are the hallmark of viral hemorrhagic diseases, involvement of blood vessels and endothelial cells in VHFs has been an active area of research. In some VHFs, such as Ebolavirus and hantavirus infection, direct infection of endothelial cells has been clearly demonstrated, while in others, such as the dengue viruses (DENV), direct infection of endothelial cells has been controversial. In addition to direct viral effects, mediators produced by the host innate immune system in response to viral genetic materials and proteins and by host adaptive immune responses, play significant roles in the pathogenesis of viral hemorrhagic fevers.

Although VHFs differ significantly in various clinical aspects, a common pathological process has emerged with regard to the involvement of vascular endothelium and the process leading to the perturbation of vascular barrier integrity and coagulation abnormalities. Except for dengue, which is endemic in many parts of the world with frequent major outbreaks, most other VHFs occur sporadically as isolated outbreaks. As such, detailed information about pathogenesis is lacking for many VHFs. In this review, we will focus on 2 well-characterized but distinct VHFs: dengue virus and hantavirus infection. Similarities and differences between these VHFs will be discussed to highlight common pathways and mechanisms that may be shared among VHFs.

## Agents

Dengue and hantaviruses are RNA viruses belonging to the Flaviviridae and Bunyaviridae families, respectively. Other than being RNA viruses, they do not share any other molecular common features. Table 1 summarizes different aspects of dengue and hantavirus infections. Dengue viruses are a group of 4 serologically distinct RNA viruses: DENV1, DENV2, DENV3 and DENV4. Their genomes are a 10-kb, single positive-stranded RNA molecule that encodes 3 structural genes: envelope (E), capsid (C) and preM (prM) proteins; and 7 nonstructural genes: NS1, NS2A, NS2B, NS3, NS4A, NS4B and NS5 (Gubler et al. 2007). The viral proteins are made as a single polypeptide that is cleaved by both host and viral proteases, yielding individual proteins. The envelope protein binds to host cells and mediates viral uptake and entry (Kuhn et al. 2002). The differences in the amino acid sequences of envelope proteins are the basis for the serologically distinct dengue serotypes. The prM protein on immature viral particles prevents fusion of the envelope protein and cleavage of prM by a host enzyme, furin, resulting in infectious mature particles (Junjhon et al.. 2008, 2010). Interestingly, DENV-infected cells release significant amounts of immature particles that were previously believed to be non-infectious (Junjhon et al. 2008, 2010). However, recent studies have suggested that antibodies to DENV prM may facilitate uptake of immature particles into host cells and enhance viral uptake and replication during a secondary DENV infection (Dejnirattisai et al. 2010; Rodenhuis-Zybert et al. 2010). Nonstructural proteins play important roles in gene replication and protein processing. In addition, several nonstructural proteins, including NS1, NS2, NS4 and NS5, have been shown to play important roles in immune evasion and pathogenesis of dengue illness (Aguirre et al. 2012; Munoz-Jordan et al. 2005; Yu et al. 2012).

**Table 1:** Similarities and differences between dengue hemorrhagic fever (*DHF*) and hantavirus pulmonary syndrome (*HPS*) and hemorrhagic fever with renal syndrome (*HFRS*)

	DHF	HPS/HFRS
Agent	Dengue viruses	New World hantavirus (HPS) Old World hantavirus (HFRS)
Distribution	Asia, Africa, Pacific, Americas	Americas (HPS) Asia, Europe (HFRS)
Genome	10-kb positive-strand RNA	11-kb negative-strand RNA
Transmission	Mosquito bite	Aerosol from rodent excreta Limited human to human
Clinical findings	Fever, thrombocytopenia, coagulopathy, plasma leakage	Fever, thrombocytopenia, coagulopathy, plasma leakage
Sites of plasma leakage	Pleural and abdominal cavities	Lungs (HPS), kidney (HFRS)
Mortality rate	1–5 %	Up to 40 % (HPS) 5–15 % (HFRS)
Endothelial infection	Unclear (in vivo)	Severe
Endothelial pathology	Minimal	Minimal
Upregulated cytokines	IFN-γ, IL-1, IL-6, IL-8, IL-10 IP-10, MCP-1, VEGF, angiopoietin 2	IFN- γ IL-6, IP-10, MCP-1, VEGF
Increased risk of severe disease with a previous exposure	Yes	No
Enhanced immune activation in severe disease	Yes	Yes

Hantaviruses are a group of more than 30 related viruses belonging to the Bunyaviridae family. They are broadly classified into Old World and New World hantaviruses, according to geographical distribution (Hjelle and Torres-Perez 2010; Ksiazek et al. 1995; Morzunov et al. 1998; Schmaljohn and Nichol 2007; Spiropoulou et al. 1994). The Old World viruses are found in Europe and Asia and cause hemorrhagic fever with renal syndrome (HFRS), while the New World viruses are found in the Americas and cause hantavirus pulmonary syndrome (HPS).

Hantaviruses are enveloped RNA viruses with a 3-segment, negative-sense RNA genome (Schmaljohn and Nichol 2007). The term “negative-sense” refers to an RNA genome complementary to the messenger RNA that encodes the viral proteins. The RNA segments are designated as small (S; 1,600–2,060 nt), medium (M; ∼3,700 nt) and large (L; 6,500–7,000 nt). The S segment encodes the nucleocapsid (N) protein, the L segment, the polymerase L protein, the M segment and the 2 glycoproteins, Gn and Gc. The naked RNA genome is not infectious; an infectious viral unit requires the viral RNA to be encapsidated by N to form the nucleocapsid core and the presence of the L polymerase protein. Both N and the 2 glycoproteins have been implicated in suppressing the innate immune response during early infection (Macneil et al. 2011). Some hantaviruses also encode a single NS protein (NSs) in an overlapping second open reading frame within the N coding region (Jaaskelainen et al. 2007; Plyusnin 2002; Spiropoulou et al. 1994). The role of NSs has not been precisely defined but no apparent link between NSs expression and hantavirus pathogenicity has been found.

## Disease transmission and clinical manifestations

DENV and hantaviruses are quite distinct in their mode of transmission. DENV are transmitted by mosquito vectors, *Aedes aegypti* and, to a lesser extent, *Ae. albopectus*, while hantaviruses are transmitted by inhalation of infectious rodent excreta (Gubler et al. 2007; Schmaljohn and Hjelle 1997). Changes in local weather patterns have been known to be associated with HPS outbreaks due to increases in rodent vector populations (Nichol et al. 1993; Schmaljohn and Hjelle 1997; Schmaljohn and Nichol 2007). In addition, hantaviruses have been isolated from other mammals, including shrews, moles and bats and these animals may also serve as viral reservoirs. Behavioral and ecological factors play important roles in the transmission of DENV. Increased mosquito vector populations associated with rapid urbanization have been considered an important factor contributing to severe dengue illness outbreaks in Southeast Asia after the Second World War (Gubler 1978). In addition, changes in global weather patterns and increased human mobility will likely further expand the areas affected by DENV.

DENV and hantavirus-caused illnesses share early clinical presentations common to many viral infections: fever, myalgia, headache, nausea, vomiting and abdominal pain. Maculopapular skin rash may be found in dengue illness and helps in differential diagnosis against respiratory viral infections. After 4–5 days of the prodromal phase, vascular leakage, thrombocytopenia and coagulation abnormalities occur. The locations of vascular leakage dictate the clinical manifestations of these diseases. Plasma leakage in DENV infection occurs in the pleural and abdominal cavities and in severe cases may lead to volume depletion (Nimmannitya 1993; Trung and Wills 2010). In HPS, lung vessels are affected, resulting in pulmonary edema and respiratory failure (Boroja et al. 2002; Castillo et al. 2001; Duchin et al. 1994; Knust et al. 2012). With proper supportive treatment, patients who survive this phase usually rapidly convalesce without long-term sequelae. Infections with Old World hantaviruses and Andes virus (ANDV) cause vascular leakage in the kidneys, manifested as proteinuria and hematuria that may lead to acute renal failure. Similarly, the acute phase (or hypotensive phase) is followed by a complete recovery in most cases.

Not all infected individuals experience severe disease. In fact, asymptomatic and undifferentiated febrile illnesses are common. This is particularly true for DENV infections. In highly endemic areas, such as Southeast Asia, a primary infection occurs early in life and is usually asymptomatic. Subsequent infections with another serotype of DENV are common, as the neutralizing antibodies to the primary infecting virus do not effectively protect against other serotypes (Halstead 2007). Secondary infections and primary infections in older children and adults are associated with more severe manifestations. Clinical dengue illness is classified into dengue fever (DF) and dengue hemorrhagic fever (DHF). By definition, plasma leakage only occurs in DHF (Nimmannitya 1993). Hemorrhagic manifestations occur both in DF and DHF but tend to be more severe in the latter. In the past few years, another clinical case classification scheme has been proposed, which classifies illnesses into dengue and severe dengue (World Health Organization 2009). Severe dengue is defined as dengue with (1) plasma leakage leading to shock, (2) severe organ impairment and (3) severe hemorrhage. As this new classification is geared towards classifying cases based on late and severe outcomes, which may reflect combinations of factors not related to the underlying pathology, its applicability for pathogenesis research may be limited.

A number of host factors associated with severe disease in dengue and HPS have been reported. Females tend to more severely affected by both infections (Guerra-Silveira and Abad-Franch 2013; Hjertqvist et al. 2010; Klein et al. 2011). Host genetic polymorphisms at HLA and tumor necrosis factor-α (TNF-α) gene loci that are associated with disease severity have been reported in both conditions (Borges et al. 2010; Stephens 2010; Stephens et al. 2002). The HLA association is indicative of the role of T cell-mediated mechanisms in pathogenesis. Other dengue illness severity-related polymorphisms include DC-SIGN (CD209), transporters associated with antigen presentation (TAPs), Fc receptor, cytotoxic lymphocyte antigen 4 (CTLA4), mannose binding lectin-2 (MBL-2) and cytokine genes TNF-α, and TGF-β (Acioli-Santos et al. 2008; Perez et al. 2010; Sakuntabhai et al. 2005).

## Pathology

Limited human autopsy studies have been performed in dengue cases. The most often observed histological changes are tissue edema and mild mononuclear cell infiltration (Bhamarapravati et al. 1967; Fresh et al. 1969). Hemorrhages, both microscopic and gross bleeding, are common findings in fatal dengue. The changes in vascular morphology are generally unremarkable. Limited apoptosis of endothelial cells has been reported in one study (Limonta et al. 2007). The extent of hepatocellular injury varies among studies. No definitive study has characterized infiltrating inflammatory cells in tissues. The primary pathology of HPS is pulmonary edema. Accumulation of fluid in the alveoli and mild to moderate interstitial infiltration with mononuclear cells have been observed. Similar to dengue, morphologic changes of the endothelium are uncommon, consisting primarily of swollen endothelial cells (Zaki et al. 1995; Zaki et al. 1996). In both diseases, proliferation of lymphoid cells in the spleen and lymph nodes is commonly observed.

Antigen staining and viral gene detection have demonstrated that endothelial cells of various organs can be infected with hantaviruses (Nolte et al. 1995; Wang et al. 1997; Zaki et al. 1995). The extent of viral infection of endothelial cells correlates with disease severity. As indicated above, no morphological changes were observed despite heavy infection. Lung epithelial cells were not infected by hantaviruses and appeared intact in HPS. In HFRS, however, viruses have been identified in tubular cells of the kidneys (Groen et al. 1996). Other cells in which hantaviruses have been located include follicular dendritic cells (FDC) and monocytes (Green et al. 1998; Groen et al. 1996; Zaki et al. 1995). Studies of human tissue from fatal dengue cases and from small animal models have shown that monocytes, tissue macrophages and lymphocytes express antigen and contain viral RNA (Balsitis et al. 2009; Jessie et al. 2004). In contrast to hantaviruses, DENV have not been definitely identified in endothelial cells. Although lung alveolar endothelial cells and liver sinusoidal cells stain positive for DENV antigen, in situ hybridization did not demonstrate viral RNA in these cells (Jessie et al. 2004).

## Virus–host interaction and innate immune response

Virus interaction with host cell surface molecules results in viral uptake and activation of the innate immune system. Several molecules have been shown to interact with DENV. Among these, the best characterized are 2 lectin molecules, CD209 and CLEC5A (Chen et al. 2008; Tassaneetrithep et al. 2003). Both molecules bind DENV particles but CD209 probably plays a role in viral uptake, while binding of DENV to CLEC5A results in production of pro-inflammatory cytokines. Viral RNA is also detected in various cellular compartments by TLRs and intracytoplasmic sensors, such as RIG-I and MDA-5 (Nasirudeen et al. 2011; Surasombatpattana et al. 2011). Activation of these pathways leads to production of type I interferons (IFN) and proinflammatory cytokines, including IL-6, IL-8 and other chemokines. Expression of certain DENV gene products, such as NS5 and NS4, also activates production of pro-inflammatory cytokines through the NF-κB pathway in various cell types, including lung epithelial cells, monocytes and human umbilical vein endothelial cells (HUVEC) (Bosch et al. 2002; Kelley et al. 2012; Medin et al. 2005). Mediators produced by these cells may play a role in altered vascular permeability in dengue.

Hantaviruses use β3 and β1 integrins and CD55 as their cellular receptors (Gavrilovskaya et al. 1998). Interaction between hantaviruses and β3 integrins has been implicated in altered permeability by enhancing the response to vascular endothelial growth factor (VEGF) (Gorbunova et al. 2010). Pathogenic hantaviruses have developed multiple mechanisms to evade the early host innate immune response and inhibit the induction of type 1 IFN in infected cells, allowing efficient viral replication and spread. One way in which hantaviruses avoid activating type I IFN pathways is by using a prime and realign transcription mechanism that results in viral RNA transcripts possessing a terminal 5′ monophosphate instead of 5′ triphosphate (Garcin et al. 1995; Habjan et al. 2008; Handke et al. 2009). In addition, hantavirus glycoproteins and N protein can inhibit IFN signaling, either by directly inhibiting RIG-I and IRF-3 activation or by inhibiting STAT1 and STAT2 phosphorylation and nuclear translocation (Alff et al. 2008; Levine et al. 2010; Spiropoulou et al. 2007). The N protein has also been implicated in inhibiting inflammatory responses by blocking the import of NF-κB to the nucleus (Taylor et al. 2009a). This inhibition seems to be characteristic of the Old World pathogenic hantaviruses (Taylor et al. 2009b).

Hantaviruses can infect a variety of cell types, including endothelial cells, tissue macrophages, dendritic cells (DC) and renal tubular cells (Krautkramer et al. 2011; Markotic et al. 2007; Pensiero et al. 1992; Raftery et al. 2002). Infection of endothelial cells induces production of numerous cytokines, including monocyte chemoattractant protein (MCP-1), regulated upon activation, normal T cell expressed and secreted (RANTES) and IFNγ-induced protein 10 (IP-10) (Sundstrom et al. 2001). Primary human DC infected with hantaviruses in vitro are quickly activated and secrete pro-inflammatory cytokines like TNF-α and an active form of matrix metalloprotease 9 (MMP-9) (Marsac et al. 2011; Raftery et al. 2002). In both DENV and hantavirus infections, DC and monocytes/macrophages are possibly the initial sites of viral replication, contributing to the subsequent viremia and infection of other cell types. Based on available in vivo data, infection of other cell types, including endothelial cells, seems to be limited in DENV infection. In contrast, exuberant infection of endothelial cells is typical in hantavirus infections (Zaki et al. 1995). The interaction between host cells and the pathogens and their products induces both an antiviral response through type I IFN and a pro-inflammatory response through various cytokines and chemokines. These mediators are not only critical in elaborating antigen-specific adaptive immunity but also directly contribute to endothelial cell activation, leading to integrity perturbation and coagulopathy observed in these VHFs.

## Direct effects of DENV and hantavirus on endothelial cells

The established paradigm proposes that vascular endothelium dysfunction upon hantavirus and DENV infection is based largely on immune modulation. However, recent studies have suggested that direct viral effects may contribute to altered endothelial functions. These studies are based on the findings that virus infection alone can cause a loss of integrity of the vascular endothelium by causing secretion of permeability factors. Infection of human primary lung endothelial cells with HPS-associated hantaviruses has resulted in increased permeability and loss of endothelial cell barrier integrity (Shrivastava-Ranjan et al. 2010). VEGF-R2 and VE-cadherin maintain the endothelial cell barrier (Lampugnani et al. 2006). VEGF binding to VEGF-R2 causes its activation and dimerization, thereby initiating the internalization and degradation of VE-cadherin and disruption of adherens junctions (Gavard and Gutkind 2006; Xiao et al. 2003). Consistent with this process, antibody blockage of VEGF-R2 activation inhibited hantavirus-induced VE-cadherin reduction. However, in a recently developed in vitro capillary blood vessel model, in which endothelial cells and vascular smooth muscle cells were co-cultured, secreted VEGF was detected but VE- cadherin degradation was not observed (Taylor et al. 2013). This model demonstrated, instead, a role for bradykinin, a potent inflammatory peptide, in hantavirus-induced capillary leakage. It is possible that both VEGF and bradykinin could work synergistically to increase vascular permeability in hantavirus-infected cells.

The most prominent cellular response to hantavirus infection is induction of hyper-permeability of the microvascular endothelium. This has been shown initially by the exogenous addition of TNF-α to hantavirus-infected endothelium (Niikura et al. 2004). More recently, a number of in vitro studies have shown that adding VEGF to primary endothelial cell monolayers infected with pathogenic hantaviruses activates VEGF-R2 and Src kinase, leading to VE-cadherin internalization and degradation and induction of hypermeability (Gavrilovskaya et al. 2008; Gorbunova et al. 2010, 2011). Adding factors like angiopoietin-1 and sphingosine-1-phosphate (S1P), which inhibit VEGF-R2 induced permeability, blocked VE-cadherin internalization in response to VEGF (Gavard and Gutkind 2006; Gavrilovskaya et al. 2008; Gorbunova et al. 2011).

In contrast to hantaviruses, DENV infection of the vascular endothelium has not been well documented in vivo. DENV antigen has been identified in endothelial cells in the lungs, liver and spleen in humans and experimental mouse models. However, conclusive evidence of DENV infection in human endothelial cells is still lacking. Histopathology studies of fatal dengue cases have demonstrated cell edema and limited pyknosis. In vitro, DENV can infect endothelial cells, resulting in the production of chemokines, including IL-8, RANTES and monocyte chemoattractant protein-1 (MCP-1). Additionally, increased cell surface expression of ICAM-1, β3 integrin and VEGF-R2 has been reported (Srikiatkhachorn et al. 2007; Zhang et al. 2007). Studies examining direct effects of DENV on endothelial permeability have shown conflicting results. Elevated circulating levels of soluble surface molecules expressed by endothelial cells, such as soluble intercellular adhesion molecule (sICAM-1) and vascular cell adhesion molecule (sVCAM-1), have been reported and are indicative of in vivo activation of endothelial cells. In addition to direct viral effects, endothelial permeability can also be altered by interactions between DENV-infected monocytes and dendritic cells via effects of TNF-α and metalloproteinase enzymes secreted by these cells (Carr et al. 2003; Kelley et al. 2012; Lee et al. 2006; Luplertlop et al. 2006).

## The role of adaptive immunity

The role of adaptive immunity in disease progression has been demonstrated in both DENV- and hantavirus-induced human diseases. This adaptive immunity is characterized by the activation of antigen-specific CD4+/CD8+ T cells and B cells. A robust T cell response is generated in humans during hantavirus infection and is followed by a long-lived memory T cell response (Van Epps et al. 2002). Several CD4+ and CD8+ T cell clones obtained from the blood of acute and convalescent HPS patients recognize epitopes from N, Gn and Gc viral proteins (Ennis et al. 1997; Kilpatrick et al. 2004; Terajima et al. 2004; Van Epps et al. 1999). Higher frequencies of Sin Nombre virus-specific CD8+ T cells have been detected in patients with severe HPS than in patients with less severe symptoms (Kilpatrick et al. 2004). These are all strong indications that virus-specific CD8+ T cells play a key role in virus-induced immunopathology (Terajima and Ennis 2011). Other studies indicate that long-lived effector memory T cell responses may contribute to protective immunity in ANDV-infected patients (Manigold et al. 2010; Prescott et al. 2013). T cell depletion has no effect on disease outcome in the hamster model of HPS (Hammerbeck and Hooper 2011; Prescott et al. 2013). This argues that induction of HPS, at least in the hamster model, is not based on virus-induced immune modulation. Additionally, NK cells are rapidly expanded and remain elevated for a considerable time after human hantavirus infection (Bjorkstrom et al. 2011).

Hantaviruses induce a long-lasting humoral immune response (Settergren 1991). Antibodies to hantaviral N protein are present in the serum of patients soon after the onset of disease, quickly followed by neutralizing antibodies directed against viral glycoproteins (Jenison et al. 1994; Lundkvist et al. 1997; Valdivieso et al. 2006). High titers of neutralizing antibodies in the patient serum correlate with milder disease outcomes (Bharadwaj et al. 2000; MacNeil et al. 2010).

The role of adaptive immunity in the pathogenesis of dengue is indicated by the increased risk of severe disease during a secondary DENV infection. Enhanced viral uptake and replication by cross-reactive non-neutralizing antibodies specific to the previously exposed DENV structural proteins, such as prM or E proteins, may lead to increased antigen load and enhanced immune activation. Consistent with this notion, higher levels of viremia and viral RNA have been associated with DHF (Libraty et al. 2002a, b; Srikiatkhachorn et al. 2012; Vaughn et al. 2000). Further, studies have demonstrated higher frequencies of DENV-specific T cells (quantified using tetramer staining and functional assays) and increased expression of activation markers in DHF in comparison to DF (Green et al. 1999a; Mongkolsapaya et al. 2003). Enumeration of DENV-specific T cells using HLA-peptide tetramer has revealed that CD8+ T cells specific to the newly exposed DENV and to previously exposed DENV are expanded during a secondary infection. Interestingly, cross-reactive memory T cells were preferentially expanded during a secondary infection. Functional analyses of these cross-reactive T cells have demonstrated distinct patterns of cytokine production upon activation by epitope peptides corresponding to the amino acid sequences found in the infecting or previously exposed DENV (Bashyam et al. 2006; Friberg et al. 2011; Mangada and Rothman 2005). Ex vivo analyses of T cells from dengue patients revealed decreased expression of degranulation marker (a marker for cytolytic activity) and increased cytokine production when cross-reactive memory T cells were stimulated with epitope peptides of the infecting DENV serotype (Mongkolsapaya et al. 2006). The decreased cytolytic activity and enhanced cytokine response may play a critical role in the loss of vascular integrity, leading to plasma leakage.

DENV-specific antibodies may play dual roles in protection against and exacerbation of severe disease. Serotype-specific antibodies have long been thought to be important in protection against subsequent infections. Higher neutralizing antibody titers were associated with milder disease in subsequent infections in a cohort study (Endy et al. 2004). However, the lack of protection despite the presence of neutralizing antibodies demonstrated in a recent vaccine trial has called into the question the role of neutralizing antibodies and the ways by which these antibodies are measured; it is unclear whether they reflect protective immunity (Sabchareon et al. 2012). As previously stated, sub-neutralizing levels of antibodies against DENV envelope protein and antibodies to prM could enhance viral uptake and replication in vitro. In addition, a series of studies have shown that DENV-specific antibodies exhibited cross-reactivity against a number of host antigens, including H^+^-transporter/ATP synthase, protein disulfide isomerase (PDI), vimentin, heat shock protein 60, fibrinogen and plasminogen (Chuang et al. 2011; Lin et al. 2011; Liu et al. 2011) and have been implicated in the pathogensis of dengue by inducing abnormal activation and functions of platelets and endothelial cells, leading to endothelial cell apoptosis and hemorrhage in experimental animals (Falconar 1997; Sun et al. 2007). The role of these antibodies in the pathogenesis of human dengue infection is unclear, since autoimmune manifestations are not observed after recovery from dengue, when these antibodies should persist beyond the acute illness period.

## Cytokines and angiogenic factors relevant in VHFs

High levels of cytokines have been detected in the plasma and serum or pleural effusions of patients infected with hantavirus or DENV (Borges et al. 2008; Mori et al. 1999). DENV-infected monocytes and DC produce a number of mediators, such as IL-6, IL-8 and IFN-γ-inducible chemokines CXCL9, CXCL10 and CXCL11, all of which have permeability-enhancing and chemoattractant properties (Bosch et al. 2002; Dejnirattisai et al. 2008). Immunomodulatory cytokine IL-10 can be produced by DENV-infected monocytes and DC and elevated IL-10 levels have been reported in DHF patients (Green et al. 1999b; Ubol et al. 2010). One cytokine of special interest is TNF-α, a permeability-enhancing and pro-coagulation factor. While it has been extensively studied, reports on TNF-α levels in severe DENV infection are conflicting (Azeredo et al. 2006; Braga et al. 2001). Enhanced activation of DENV-specific T cells likely contributes to the elevated cytokine levels in DHF. Multiple cytokines are secreted by DENV-specific T cells, including Th1 type cytokines IL-2, IFN-γ, TNF-α and various chemokines (Bashyam et al. 2006; Mangada and Rothman 2005). Indeed, elevated levels of both Th1-type (IFN-γ) and Th2-type cytokines (IL-4, IL-13, IL-10) have been reported in DHF (Bozza et al. 2008; Butthep et al. 2012). Primary human monocytes and DC infected with hantaviruses in vitro are quickly activated and secrete a number of pro-inflammatory cytokines, including TNF-α (Cebalo and Markotic 2007; Markotic et al. 2007; Marsac et al. 2011; Raftery et al. 2002). In vitro studies have also shown the induction of chemokines, such as MCP-1, RANTES and IP-10, in hantavirus-infected vascular endothelium (Geimonen et al. 2002; Khaiboullina and St Jeor 2002; Sundstrom et al. 2001). These chemokines can recruit immune cell infiltrates into the lung and other organs. Based on immunocytochemical studies, several cytokines have been detected in patients with severe hantavirus disease; these cytokines are potentially produced by T cells and include TNF-α, IL-2, IL-6 and IFN-γ (Mori et al. 1999; Temonen et al. 1996). Both TNF-α and IL-2 can increase vascular permeability.

Changes in circulating levels of angiogenic factors, including VEGF, its soluble receptors VEGF-R1 and VEGF-R2 and angiopoietin-1 and angiopoietin-2 have been reported to correlate with DENV disease severity (Michels et al. 2012; Srikiatkhachorn et al. 2007). Notably, increased VEGF-R2 expression has been associated with an enhanced response to VEGF by DENV-infected endothelial cells (Srikiatkhachorn et al. 2007). In support of this notion, a similar pro-leakage angiogenic profile of elevated levels of angiopoietin-2 and low levels of angiopoietin-1 has been seen in other conditions with plasma leakage, such as sepsis and correlates with severity and outcomes (Parikh et al. 2006; Ricciuto et al. 2011). Increased levels of free VEGF have also been reported in sera from hantavirus patients (Gavrilovskaya et al. 2012; Ma et al. 2012; Shrivastava-Ranjan et al. 2010) and low levels of angiopoietin-1 have been seen in HFRS patient sera (Hwang et al. 2009). Endothelial cell activation, marked by elevated levels of circulating soluble surface molecules expressed by endothelial cells, such as soluble intercellular adhesion molecule 1 (sICAM-1) and soluble vascular cell adhesion molecule (sVCAM-1), has been detected in both DHF and HFRS human sera (Han et al. 2010).

The contribution of these permeability-regulating cytokines in the pathogenesis of hantavirus disease and DHF is not clear. The conflicting results in cytokine level data in hantavirus and DENV infection may be due to the heterogeneity in study designs, times of sample collection, differences in patient populations and clinical classification. An additional difficulty in interpreting studies of hantaviral diseases, particularly HPS, is the small number of samples available for such analysis. The roles of these mediators in the manifestations of these infections can only be definitely tested in animal models. Notably, a study using a mouse model of DENV infection has demonstrated that in vivo neutralization of TNF-α prevented death in this model. Whether this is relevant to human dengue illness is not clear, since these small animal models do not faithfully reproduce the clinical picture of human DHF.

## Endothelial cell-based therapeutic intervention

Considering that microvascular leakage is a common factor that correlates with disease severity for both hantaviruses and DENV, it is reasonable to assume that treatments that block vascular permeability could be used to treat the diseases caused by these viruses. Several agents that enhance endothelial barrier integrity have been used in tissue culture experiments of hantavirus infections. Some of these agents are small molecule anti-angiogenic agents, like Pazopanib and Dasatinib, which prevent ANDV-induced hyper-permeability by blocking VEGF-R2 or Src kinase (Gorbunova et al. 2011). An alternative approach is to strengthen the infected vascular barrier by activating Roundabout homolog 4 (Robo-4)-dependent signaling pathways using the soluble ligand Slit-2 (Gorbunova et al. 2013). Based on recent reports, small molecules preventing activation of the plasma kallikrein–kinin system and bradykinin release could also be used to treat hantavirus diseases (Taylor et al. 2013).

Studying the importance of vascular leakage in hantavirus and DENV infections, as well as determining the efficacy of anti-leakage treatments for these diseases, would be facilitated by the use of animal disease model experiments. The absence of appropriate animal models mimicking human DENV disease progression is a major limitation of such studies. Recent studies using immunocompromised mice and/or mouse-adapted DENV have implicated TNF-α in mediating plasma leakage and hemorrhage, possibly through the production of reactive oxygen species (Shresta et al. 2006; Wu-Hsieh et al. 2009). Currently, the only small animal model for hantavirus disease is infection of Syrian golden hamsters with two South America hantaviruses, ANDV and Maporal virus (Hooper et al. 2001; Milazzo et al. 2002). Infecting hamsters with these viruses results in disease with characteristics similar to those of human HPS, including interstitial pneumonitis and microvascular leakage. No small animal model exists for HFRS, though HFRS-like illness has been observed in cynomolgus macaques infected with a Puumala virus strain that was propagated in the rodent host and not tissue culture-adapted (Klingstrom et al. 2002; Sironen et al. 2008). Currently, in vivo experiments using small molecule tyrosine kinase inhibitors have been initiated in hantavirus-infected hamsters (Dolgin 2012). Of note, a successful treatment of one patient with a single dose of icatibant, a bradykinin receptor antagonist, has been recently reported (Antonen et al. 2013).

## Conclusion and perspectives

**Fig. 1:**
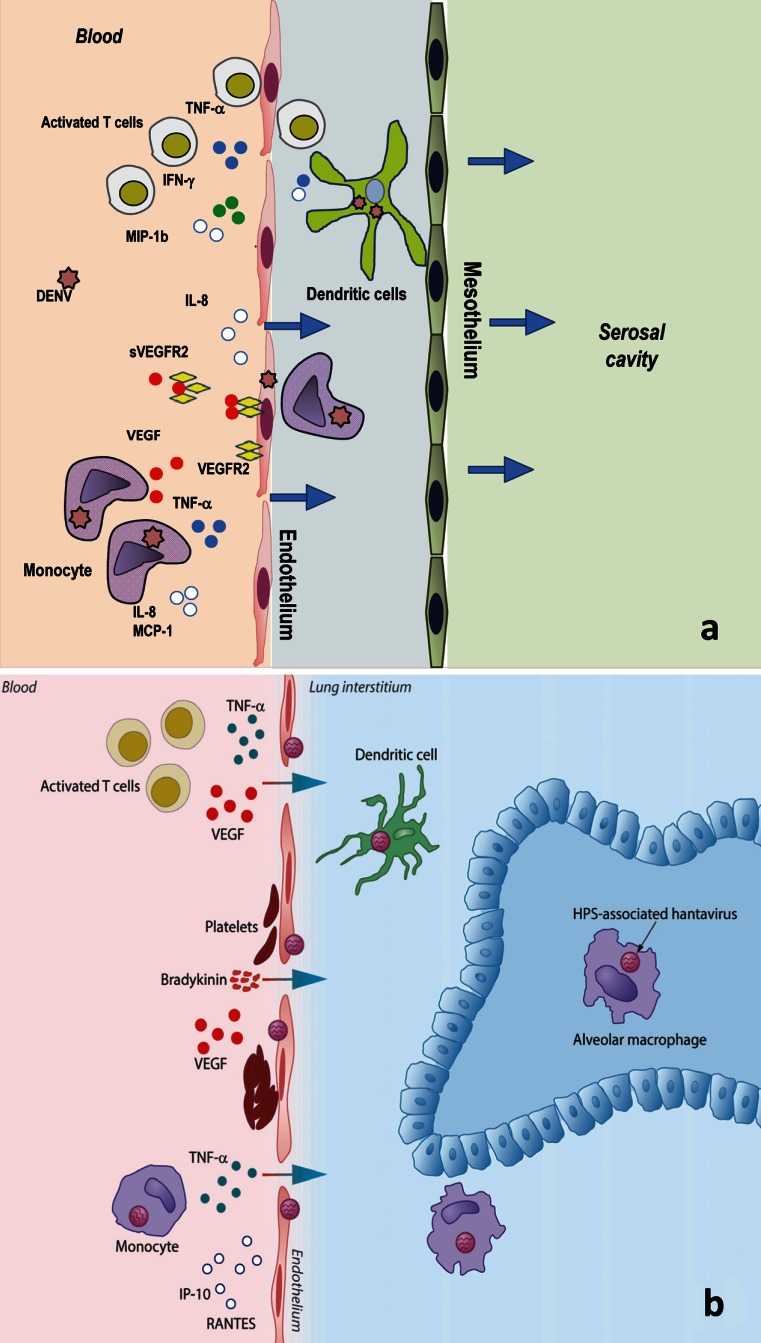
Possible mechanism of plasma leakage in DHF and HPS. **a** DENV-infected monocytes and dendritic cells release cytokines, such as TNF-α, and chemokines (IL-8, MCP-1), resulting in increased vascular permeability and recruitment of inflammatory cells. Activated T cells may release permeability enhancing mediators, including IL-2, IFN-γ, TNF-α, and the chemokines. Elevated VEGF levels may be secondary to VEGF produced by monocytes, T cells, or endothelial cells. In addition, infection of endothelial cells has been shown to suppress the production of soluble VEGFR2 and to increase surface receptor expression, leading to enhanced responsiveness to VEGF. Other potential mechanisms, not depicted, include deposition of antigen-antibody complexes and complement activation on endothelial cell surface, and autoantibodies that react to endothelial cells. The net effect is the leakage of albumin-rich fluid into serosal cavities. **b** Hantavirus accesses the vascular endothelium via infected DC and/or infected alveolar macrophages. Early post-infection, the virus inhibits induction of type 1 IFN in infected cells, allowing efficient viral replication and spread. Hantavirus-infected endothelial cells produce proinflammatory cytokines and chemokines, and upregulate adhesion molecules on their cell surface, attracting monocytes, macrophages, and T cells. In addition, the infected endothelium can release bradykinin, which can directly increase vascular permeability. Binding of the virus to β3 integrins on endothelial cells, along with the early secretion of VEGF, could trigger the disruption of adherens junctions and downregulation of VE-cadherin. Accumulation of hantavirus-infected monocytes, macrophages, and T cells in the vicinity of the endothelium can result in exuberant release of cytokines, chemokines, and permeability factors like TNF-α and VEGF. Additional VEGF could be secreted by ANDV-activated platelets. TNF-α and VEGF from these sources could reach high concentrations in the microvasculature of the lung, resulting in vascular hyper-permeability and leakage

Although distinct vascular beds are affected in DHF and HPS, leading to different clinical manifestations, these two VHFs share certain clinical features and pathophysiologic processes. Endothelial cell activation and dysfunction, perturbation of barrier integrity and activation of coagulation pathways are common in both diseases. In both conditions, the complex interplay between the viruses, the immune systems and the endothelial cells determines the activation of endothelial cells and the functional consequences (Fig. 1). Insights into the common and distinct molecular mechanisms of these conditions will be important for the development of treatment and preventive measures against them.

Due to the lack of animal models, the understanding of disease mechanisms in dengue illness and HPS has been largely derived from human studies. However, conflicting findings are common. The dynamic patterns of cytokines/biological mediators relative to the clinical course require that sample collection be performed at certain time points and the findings interpreted in the context of the clinical course. In addition, differences in sample collection methods can affect the levels of mediators in these samples, resulting in inconsistent findings. Small numbers of cases (in the case of HPS) and the limited sample volume, particularly from children infected with DENV, are additional limitations in human studies. Despite these limitations, well-planned prospective studies remain necessary in an effort to gain further insights into the pathogenesis of these diseases. The recent development of small animal models for both dengue VHF and HPS is an important advance. However, further improvement in these models to more closely mimic human diseases will be needed. Nevertheless, such animal models will be critical in the evaluation of therapeutic measures to ameliorate severe clinical manifestations, particularly plasma leakage and hemorrhage, the major causes of VHF fatality.
